# Development and Piloting of Education and Assessment of Person-Centered Care Standards

**DOI:** 10.1093/geroni/igaf122.555

**Published:** 2025-12-31

**Authors:** Tara Cortes, Shih-Yin Lin, Elizabeth Seidel

**Affiliations:** New York University/HIGN, New York, New York, United States; New York University Rory Meyers College of Nursing, New York, New York, United States; New York University Rory Meyers College of Nursing, New York, New York, United States

## Abstract

To further develop a tool that assesses the use of person-centered care (PCC) practices in long-term care (LTC) communities, the preliminary standards developed by the University of Maine in Years 1 and 2 were compared against focus group and survey findings from 5 new LTC communities across the United States conducted by the HIGN team at NYU Meyers. The preliminary standards were revised by consolidating overlapping, creating new standards, and reducing the reading level. For each of the standards, person-centered best practices were transformed into corresponding items in an organizational self-assessment questionnaire, along with a description of appropriate supporting documents for corroboration. The organizational assessment items to evaluate each standard were developed iteratively during weekly team meetings, in consultation with an expert panel consisting of national advocates for aging research and long-term care. Companion educational modules and booklets were created to familiarize care communities with the 7 person-centered domains, the standards, the associated behaviors, and supporting documents including policies within each domain. Findings from this research can inform the development and implementation of best practices in LTC settings. Future research should (1) evaluate comprehension of this assessment tool among LTC staff via cognitive interviewing and (2) pilot this tool along with accompanying educational materials in a larger sample of communities across the United States to ensure generalizability. The tools developed in this project can be used to evaluate person-centeredness across long-term care settings.

